# MPBind: a multitask protein binding site predictor using protein language models and equivariant GNNs

**DOI:** 10.1093/bioinformatics/btaf589

**Published:** 2025-10-24

**Authors:** Yanli Wang, Frimpong Boadu, Jianlin Cheng

**Affiliations:** Department of Electrical Engineering and Computer Science, University of Missouri, Columbia, MO 65211, United States; NextGen Precision Health Institute, University of Missouri, Columbia, MO 65211, United States; Department of Electrical Engineering and Computer Science, University of Missouri, Columbia, MO 65211, United States; NextGen Precision Health Institute, University of Missouri, Columbia, MO 65211, United States; Department of Electrical Engineering and Computer Science, University of Missouri, Columbia, MO 65211, United States; NextGen Precision Health Institute, University of Missouri, Columbia, MO 65211, United States

## Abstract

**Motivation:**

Proteins interact with a variety of molecules, including other proteins, DNAs, RNAs, ligands, ions, and lipids. These interactions play a crucial role in cellular communication, metabolic regulation, gene regulation, and structural integrity, making proteins fundamental to nearly all biological functions. Accurately predicting protein interaction (binding) sites is essential for understanding protein interaction and function.

**Results:**

In this work, we introduce MPBind, a multitask protein binding site prediction method, which integrates protein language models (PLMs) that can extract structural and functional information from sequences and equivariant graph neural networks (EGNNs) that can effectively capture geometric features of 3D protein structures. Through multitask learning, it can predict binding sites on proteins that interact with five key categories of binding partners: proteins, DNA/RNA, ligands, lipids, and ions. MPBind generalizes across the five molecular classes with state-of-the-art accuracy, achieving AUROC scores of 0.83 and 0.81 for protein–protein and protein–DNA/RNA-binding site prediction, respectively. Moreover, MPBind outperforms both general and task-specific binding site prediction methods, making it a useful, versatile tool for protein binding site prediction.

**Availability and implementation:**

The source code of MPBind is available at the GitHub repository: https://github.com/jianlin-cheng/MPBind.

## 1 Introduction

Proteins are highly versatile biomolecules that interact with a wide range of molecules, including other proteins, DNA, RNA, ligands, ions, and lipids, to perform essential cellular functions (Berg 2003). Rather than working alone, they function as part of larger molecular machines or complexes, where their roles depend on the specific interactions they form (Zhou *et al.* 2016). These interactions are essential for key cellular processes like metabolism, DNA replication, gene expression, signaling, and structural support (Peng *et al.* 2017). A protein’s ability to bind with other molecules comes from its unique three-dimensional (3D) shape and specialized features, such as domains and motifs, which help it make precise interactions (Teichmann 2002). Understanding how proteins interact not only offers valuable insights into cellular mechanisms but also helps guide the development of therapeutic strategies by leveraging these core biological processes. With the explosive growth of protein sequences and structures, accurately predicting protein binding sites (i.e. the specific residues of a protein that interact with its partners) is essential for decoding protein interactions and their biological roles, which is important for biological research, biotechnology development, drug design, and disease treatment.

The rapid increase of experimental and predicted protein structures has created both a major challenge and an opportunity for using this structural information to represent and predict protein surface properties such as binding sites (Wei *et al.* 2024). In recent years, deep learning has significantly advanced the prediction of protein binding sites (Zhao *et al.* 2020). Deep learning models can process huge amounts of protein structure data, automatically identifying complex patterns and predicting binding sites with better accuracy than before (Zhang and Kurgan 2018, Zhao *et al.* 2020). Based on the input information used by deep learning methods, they can be largely grouped into two categories: sequence-based (Zhang and Kurgan 2018) and structure-based (Zhao *et al.* 2020) methods. Structure-based approaches typically focus on using the 3D structure of proteins to pinpoint where binding sites are located, taking advantage of the arrangement of atoms and the overall protein shape to identify potential binding sites (Zhao *et al.* 2020). In this category, deep learning methods typically represent 3D structures using grid-like or voxel-based models (Ragoza *et al.* 2017, Stepniewska-Dziubinska *et al.* 2018, Prat *et al.* 2024), as well as graph-based representations (Stärk *et al.* 2022, Tubiana *et al.* 2022, Krapp *et al.* 2023, Wei *et al.* 2003), to detect patterns and identify regions likely to interact with other molecules, such as ligands, other proteins, or DNA.

Unlike structure-based methods, sequence-based deep learning approaches predict protein binding sites using only the amino acid sequence, without leveraging the protein’s 3D structure (Zhang and Kurgan 2018). These methods rely on deep learning models to recognize patterns in protein sequences and pinpoint potential binding sites (Xia *et al.* 2021, Yuan *et al.* 2022, Liu and Tian 2023, Yuan *et al.* 2024), making them useful for proteins with no known structural data. For instance, CLAPE (Liu and Tian 2023) takes protein sequences as input to predict protein–DNA-binding sites, leveraging a pre-trained protein language model (PLM) and contrastive learning without considering 3D structural information.

As the structures of most proteins can be readily obtained by high-accuracy protein structure prediction methods such as AlphaFold (Jumper *et al.* 2021, Abramson *et al.* 2024) due to the development of deep learning methods in the field (Eickholt and Cheng 2012, Jumper *et al.* 2021), it is important to fully harness the valuable insights from both protein sequences and structures to improve protein binding site prediction. ScanNet (Tubiana *et al.* 2022) takes protein 3D structures and their corresponding sequences as input to predict protein–protein interaction interfaces, primarily emphasizing the geometric information of the structures in alignment with the sequences. However, it does not fully exploit sequence information from pre-trained PLMs. DeepProSite (Fang *et al.* 2023) integrates structural and sequence information, improving predictions especially on unbound proteins, but it struggles with highly flexible regions of proteins and the increased computational complexity. GraphPBSP (Sun *et al.* 2024) leverages a hybrid deep learning framework combining graph attention and convolutional networks, but its complexity demands careful parameter tuning and can lead to overfitting, and its sensitivity to graph construction affects robustness. PeSTo (Krapp *et al.* 2023) primarily uses the atomic coordinates of protein structures as input to predict multiple types of binding sites through a geometric transformer. However, like ScanNet, it does not fully utilize sequence information in PLMs. GraphBind (Xia *et al.* 2021) leverages both protein structural information and corresponding sequence information through a hierarchical graph neural network (GNNs). However, it relies on traditional sequence alignment methods, such as HHblits profile (Remmert *et al.* 2011) and PSI-BLAST profile (Altschul *et al.* 1997), to explore sequence information, without using powerful PLMs to generate sequence features. GPSite (Yuan *et al.* 2024) takes protein sequences as input to predict multiple types of binding sites at the residue level via a graph transformer and leverages the structural information in the structures predicted by ESMfold (Lin *et al.* 2022), yielding promising results. Therefore, there is a need to fully harness the valuable insights from both protein sequences and structures to further improve protein binding site prediction.

Currently, PLMs (Elnaggar *et al.* 2022, Lin *et al.* 2023, Heinzinger *et al.* 2024), the deep learning models designed to understand and analyze 1D protein sequences in a manner similar to how natural language models process human language, have proven to be incredibly effective at analyzing and predicting protein structures, functions, and interactions. By recognizing patterns in vast amounts of protein sequence data, they can be used to identify binding sites, classify protein functions (Boadu *et al.* 2023), and even generate new protein sequences. Meanwhile, equivariant graph neural networks (EGNNs) are an emerging geometric deep learning approach suitable for protein structure analysis, as they can maintain consistent predictions when the input data undergoes transformations such as rotations or translations (Satorras *et al.* 2021, Roche *et al.* 2023). Unlike traditional GNNs such as the one used in GPSite, which operate on 2D graph structures (nodes and edges) without directly considering 3D locations, EGNNs offer an alternative, robust framework for analyzing and predicting protein structures and interactions by leveraging symmetry and 3D spatial information (Roche *et al.* 2023, Wei *et al.* 2003). Therefore, it is interesting to leverage the strengths of both PLMs and EGNNs to improve protein binding site prediction, especially for multitask predictions across different types of binding sites.

In this work, we introduce MPBind, a multitask deep learning method to predict five kinds of binding sites through one model. MPBind combines two PLMs (Elnaggar *et al.* 2022, Heinzinger *et al.* 2024), pre-trained on different datasets to provide complementary insights, with an EGNN (Roche *et al.* 2023) to capture and process both sequence and structural information. Additional information, such as secondary structures, atomic and geometric residue-level details, and geometric edge information, is also incorporated into the model. Using a multitask framework, MPBind can predict and analyze a wide range of protein binding sites, including interactions with other proteins, DNA, RNA, ligands, ions, and lipids. After training on experimental structures from the Protein Data Bank (PDB) (Berman *et al.* 2000, 2007), it can predict binding sites from either experimental structures or AlphaFold-predicted structures [e.g. ones in AlphaFold Protein Structure Database (Jumper *et al.* 2021, Varadi *et al.* 2024)].

We compared MPbind with several selected baseline models such as ScanNet (Tubiana *et al.* 2022), PeSTo (Krapp *et al.* 2023), CLAPE (Liu and Tian 2023), GraphBind (Xia *et al.* 2021), and LMetalSite (Yuan *et al.* 2022) because they represent state-of-the-art approaches employing diverse architectures—including geometric GNNs, equivariant transformers, and multi-task learning—and cover a broad range of binding site types from protein–protein to protein–metal ion interactions. Additionally, the availability of these models’ pre-trained weights facilitates a fair and direct comparison. This comprehensive benchmarking allows for a thorough evaluation of MPBind’s performance and generalizability across multiple binding categories and methodological paradigms.

## 2 Materials and methods

### 2.1 Datasets for training, validating, and testing MPBind

Two datasets are used in this work. The first dataset (Dataset1) comes from PeSTo (Krapp *et al.* 2023), which includes the protein structures released before 1 January 2022 in the PDB (Berman *et al.* 2000, 2007). Following the exact data processing procedure of PeSto, we downloaded all the protein structures from the PDB, extracted all the chains (subunits) of the structures, and clustered them based on a maximum sequence identity of 30%. Thirty percent sequence identity is a standard, stringent threshold used in the field to determine if two proteins are similar or homologous (Brenner *et al.* 1998) and remove sequence redundancy because proteins with <=30% identity likely are not evolutionarily related and do not share similar structural folds. For instance, it was used to construct the widely used UniRef30 protein sequence dataset (Suzek *et al.* 2015). The clustered chains were partitioned into training, validation, and test sets according to the exact same split used by PeSTo (Krapp *et al.* 2023) such that the proteins between different sets have less than 30% sequence identity. The three subsets are referred to as ***training_data***, ***validation_data***, and ***Test_data*,** which has 376 216 chains (∼70% of the data), 101 700 chains (∼15%), and 97 424 chains (∼15%), respectively. Because ***Test_data*** is too large for any method to run efficiently, we selected the chains from it using a threshold of a maximum of 8192 atoms (1024 × 8) and a minimum of 48 residues, resulting in 40 651 chains in total. They form a test set called ***Test1_data*** to compare MPBind and PeSTo directly. The statistics of *Test1_data* are shown in Table 1, available as supplementary data at *Bioinformatics* online, where the number of chains and binding residues for each binding type vary a lot. The uneven distribution of binding types reflects their different frequency in the non-redundant proteins in the PDB as well as the different sizes of different types of binding interfaces. Since Test1_data and the training_data splits are both randomly drawn from the same Dataset1, their binding type distributions are expected to be similar. During the training and testing, we did not try to balance the number of binding residues for each binding type because MPBind could predict each type of binding sites relatively well on the original, unbalanced data.

We also downloaded the protein structures released after 1 January 2022 (the cutoff date of Dataset1 and its subset *training_data*) and before 21 June 2024, to create the second dataset (Dataset2). The chains in Dataset2 that have >30% sequence identity with the proteins in Dataset1 were removed based on sequence alignments generated by MMseq2 (Steinegger and Söding 2017). The remaining chains were clustered using MMseqs2 at the 30% sequence identity threshold. Only one chain was selected from each cluster to form a set of test proteins, referred to as ***Test2_data***, which contains 1452 chains in total. Therefore, no chain in ***Test2_data*** has more than 30% sequence identity with any protein in Dataset1 and its subset *training_data*, ensuring it can be used to test how well MPBind can generalize to dissimilar/non-homologous proteins. Moreover, the sequence identity between any two chains in *Test2_data* is also <=30%, guaranteeing the test performance is assessed across dissimilar proteins evenly. The statistics of *Test2_data* are reported in Table 1, available as supplementary data at *Bioinformatics* online.

Since the proteins in ***Test2_data*** were released after the latest release cutoff date (1 January 2022) of the training/validation proteins of all the methods evaluated in this work and have <=30% sequence identity with any protein released before 1 January 2022 (i.e. proteins in Dataset1), it contains only new, dissimilar proteins for all the methods and can therefore serve as an objective and rigorous benchmark to evaluate their generalization capability.

To further validate and analyze MPBind, we also downloaded the 23 391 human protein structures in the human proteome from the AlphaFold-European Bioinformatics Institute (AF-EBI) database (Jumper *et al.* 2021, Varadi *et al.* 2024) and their function annotations in UniProt. This set is used to investigate how well MPBind’s predictions can be used to study biological functions.

### 2.2 Protein structure preprocessing and label extraction

Following the same procedure of PeSTo (Krapp *et al.* 2023), in a protein structure, deuterium atoms, hydrogen, water, and heavy water are all removed. Non-native molecules used to assist in solving the structures are also excluded. If an atom has alternate locations, only the first one is retained.

In a protein structure, a total of 79 kinds of molecules, including 20 standard amino acids, eight most common nucleic acids, 16 most common ions, 31 most common ligands, and four most common lipids, are selected. Two molecules are considered in contact/interaction if the minimum distance between their heavy atoms is within 5 Å. The residues in a protein chain that are in contact with other types of molecules (nucleotides, ions, ligands, lipids) or residues in other protein chains are defined as the interaction interface (binding sites). Based on the threshold, a 79 × 79 interface-type matrix is constructed for all interactions among these molecules, covering protein, DNA/RNA, ion, ligand, and lipid interactions. However, since we focus only on interactions between 20 standard amino acids (residues) of a protein and other molecules, the interface-type matrix is reduced to 20 × 79. In this matrix, entries marked with 1 indicate binding sites, while entries marked with 0 indicate non-binding sites. For a protein chain with *L* residues interacting with another molecule (a protein chain, DNA/RNA, ion, ligand, or lipid) of length *M*, there are *L × M* pairwise combinations that may or may not be in contact. Each combination is projected onto a corresponding interface-type matrix of shape 20×79, leading to a chain-specific interaction interface matrix of shape *L × M × *20 × 79, which serves as labels for training, validation, and evaluation of MPBind.

### 2.3 Feature extraction

**Figure 1.**
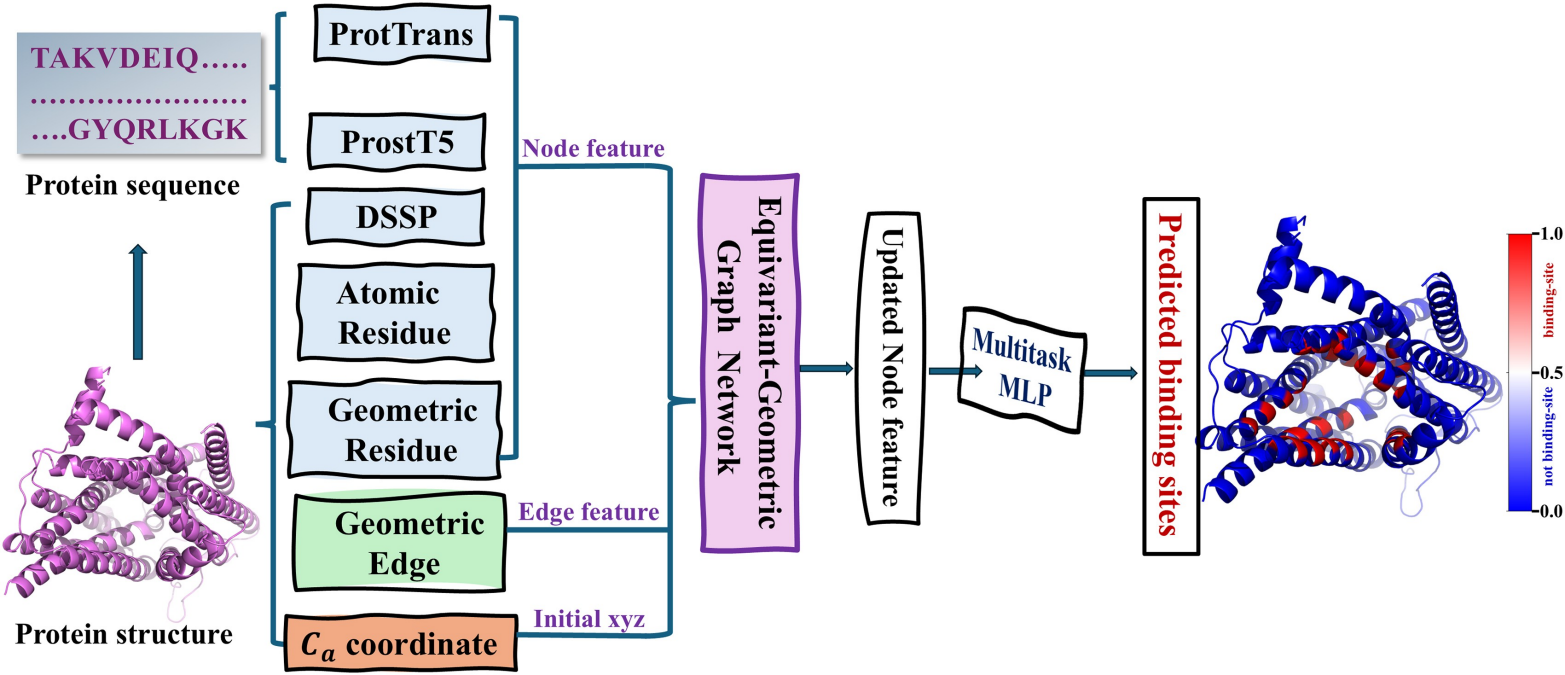
The overall architecture of MPBind. Both protein sequence and structure are used to generate node features from different perspectives. The protein sequence is processed through two PLMs, ProtTrans and ProstT5, to extract residue embeddings (a special kind of residue features). The protein structure provides secondary structure node features extracted by DSSP (Kabsch and Sander 1983, Touw *et al.* 2015), atomic residue features derived from intra-residue atomic properties, and geometric residue features obtained from intra-residue atomic coordinates. Additionally, geometric edge features are extracted from the protein structure. The five types of node features, along with the geometric edge features and the *x*, *y*, *z* coordinates of Ca atom of each node, are used as inputs to a four-layer equivariant graph neural network block to update node features. The updated node features are then passed through a multitask multi-layer perceptron (MLP) to generate a predicted score ranging from 0 to 1 for each node and each type of binding site. Each score for a node is the predicted probability that the node is a binding site of a specific type.

MPBind uses a graph to represent the input structure of a protein chain and its corresponding sequence information, where a node denotes a residue and an edge connecting two residues (nodes) if the distance between their $C_{a}$ (alpha carbon) atoms is within 15 Å. This distance cutoff threshold balances capturing meaningful spatial interactions including direct contacts and near-neighbor effects, while maintaining computational efficiency, which is consistent with prior studies employing similar thresholds (Fout et al. 2017, Ingraham *et al.* 2019, Gainza *et al.* 2020). Two types of features, node features and edge features, are extracted to represent a protein, as shown in Fig. 1.

To fully leverage the node information, we introduce five types of node features extracted from both protein sequences and structures, each of which makes a significant contribution to our model according to the ablation study. The feature extraction is described in Section 1.1, available as supplementary data at *Bioinformatics* online in detail. The dimension of each kind of node feature can be found in Table 2, available as supplementary data at *Bioinformatics* online. Finally, the five types of node features above are concatenated to form the final set of node features, resulting in an input feature tensor with size of L×2247 for an input protein with *L* residues.

To extract edge features, we treat each residue as a group of five (virtual) atoms: N, C, $C_{a}$, O, and *R*, where *R* is a virtual atom denoting the centroid of the side chain. We explore several pieces of geometric edge information, including the spatial orientation, calculated by $Q_{i}^{T}Q_{j}$, where $Q_{i}$ and $Q_{j}$ denote the coordinates of the atoms of *i*th and *j*th residues connected by an edge, the relative directions of all atoms in a neighboring residue to the $C_{a}$ atom of the residue in consideration, and the distances between atoms from two residues connected by an edge. The spatial orientation and relative direction features are the same as in GPSite.

To obtain additional geometric information about the edge, the positional embedding information is also calculated based on the index difference between two residues, using $C_{a}$ as a reference. Finally, the geometric edge features above, including orientation, direction, distance, and positional embedding information, form the final set of edge features with a size of E×450, where *E* denotes the number of edges in an input structure.

### 2.4 The architecture of MPBind

MPBind is based on an EGNN (Satorras *et al.* 2021, Du *et al.* 2022) that takes the (*x*, *y*, *z*) coordinates of the Ca atom of each node, along with node and edge features, as input. The reason for using only the position of the Ca atom of each node to represent its position in a protein structure is that the other atoms of the node have been encoded in the node and edge features. Moreover, using the coordinates of Ca atom is more computationally efficient and simpler than using all atoms of each residue.

As shown in Fig. 1, the main component of MPBind is the four-layer EGNN block, which is similar to the network used in our recent chromosome structure modeling work (Wang and Cheng 2025). Each EGNN layer updates the features of each node considering its own features, the messages from its neighboring (connected) nodes, and the relative positions between it and the neighboring nodes. The major advantage of the EGNN layer is that its feature update is equivariant to the rotation and translation of protein structure such that it can focus on extracting essential features relevant to prediction tasks while ignoring the difficulty caused by arbitrary rotation and translation of input structures. After the four EGNN layers, the updated node features are passed through a multitask multi-layer perceptron (MLP) head for each binding site type, followed by a sigmoid activation function to predict the probability that each node is a binding site for each of the five types of binding partners (proteins, DNA/RNA, ligands, lipids, and ions), respectively.

### 2.5 Training, validation, and evaluation

The graph representations of all the protein chains in the ***training_data*** dataset were used to train MPBind. The binary cross-entropy loss function was employed to measure the difference between the predicted scores and binding site labels. During training, the loss function of MPBind was minimized using the Adam optimizer with a learning rate of 0.001 to adjust its weights. The ***validation_data*** dataset was used to monitor MPBind’s performance during the training and select the best model based on the lowest validation loss. After training, the selected best MPBind model was blindly tested on two datasets, ***Test1_data*** and ***Test2_data***, for a fine-grained comparison with other methods. Finally, the selected MPBind model was used to predict the interfaces for the proteins in the human proteome dataset (Varadi *et al.* 2024), whose structures were predicted by AlphaFold2 (Yuan *et al.* 2022), for further analysis of the function implication of interface features.

In line with previous works (Xia *et al.* 2021, Krapp *et al.* 2023, Yuan *et al.* 2024), three common metrics, the area under the receiver operating characteristic curve (AUROC), the area under the precision–recall area curve (AUPRC), and accuracy (Acc) are selected to evaluate the binding site prediction for MPBind and other methods (Xia *et al.* 2021, Tubiana *et al.* 2022, Yuan *et al.* 2022, Krapp *et al.* 2023, Liu and Tian 2023). AUROC is a widely used performance metric in binary classification to assess a method’s ability to distinguish between positive and negative classes at different thresholds. AUPRC measures the alignment of predictions with the known gold standard with an emphasis on positive examples, making it a suitable evaluation metric for highly imbalanced datasets like the ones in this project, where only a small portion of residues of the proteins in the test datasets are true binding sites. It is worth noting, AUROC and AUPRC are most important metrics for evaluating the methods across the different test datasets because they are more comprehensive than the other metrics depending on a specific decision threshold.

Acc measures the proportion of correctly classified residues, meaning the number of residues that are accurately predicted as either binding or non-binding sites, over the total number of residues in the dataset, indicating the model’s overall classification accuracy, and can be calculated by the following equation:


$$\mathrm{Accuracy}\;(\mathrm{Acc})=\frac{\mathrm{TP}+\mathrm{TN}}{\mathrm{TP}+\mathrm{TN}+\mathrm{FP}+\mathrm{FN}}$$

where TP (number of true positives) refers to residues correctly predicted as binding sites, TN (number of true negatives) to those correctly predicted as non-binding sites, FP to those incorrectly predicted as binding sites, and FN to those incorrectly predicted as non-binding sites. For highly class-imbalanced datasets with only a small portion of residues with positive labels as in this work, Acc can be very high but still fail to accurately measure how a method distinguishes positive examples from negative ones. Moreover, Acc depends on the probability threshold used to decide if a residue is predicted as binding site. In this work, 0.5 is used uniformly as the decision threshold for all the methods.

## 3 Results

### 3.1 Protein–protein binding site prediction

For protein–protein interaction site (binding site) prediction, we first benchmarked MPBind alongside another multitask deep learning binding site prediction method PeSTo (Krapp *et al.* 2023) on the *Test1_data* dataset (refer to Section 2 for more details about the datasets). Both MPBind and PeSTo are general, non-task-specific prediction methods that can predict different kinds of bind sites. Both were trained on the same dataset *training_data*. The proteins in *Test1_data* have less than 30% sequence identity with those in *training_data*.

**Figure 2.**
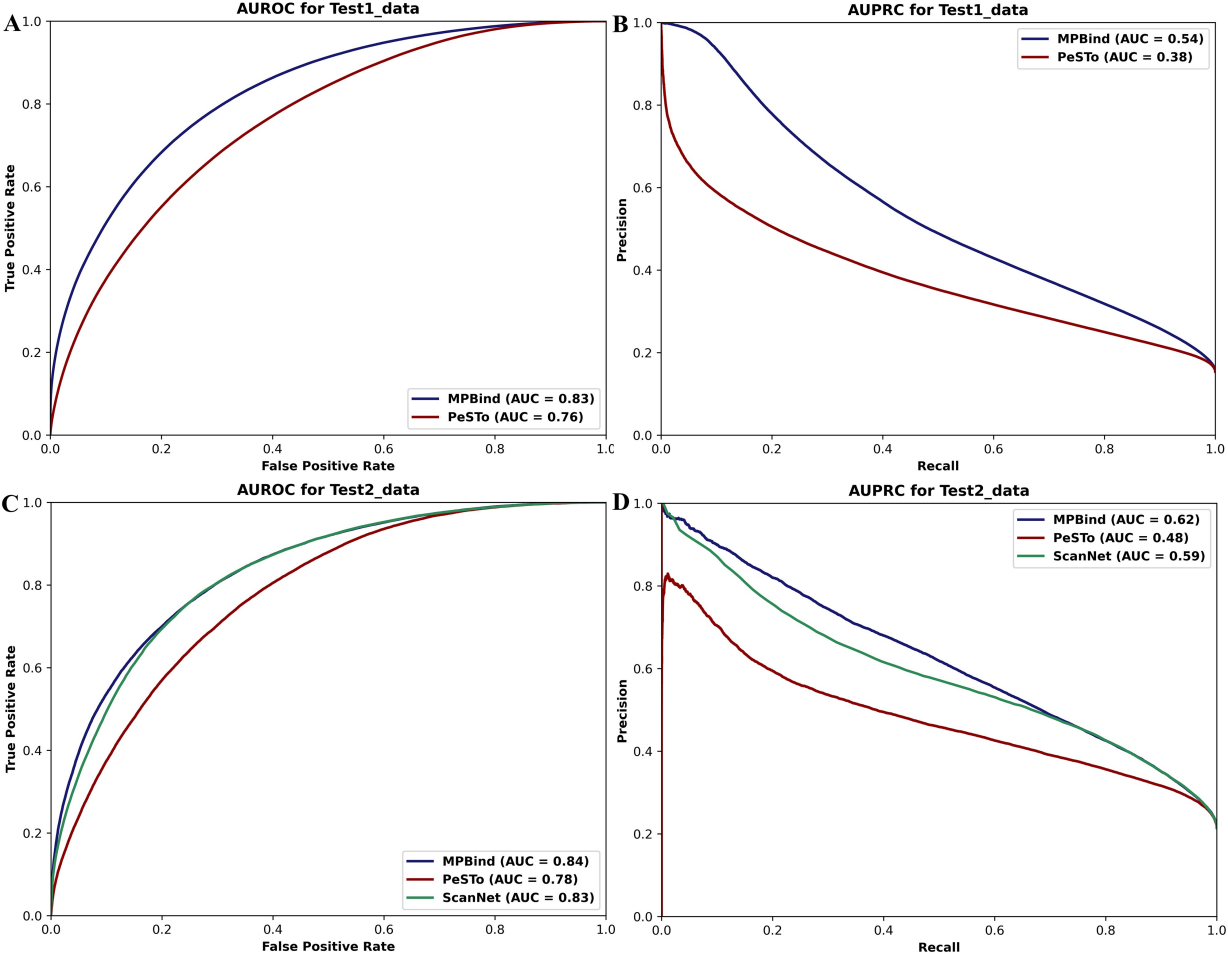
Performance comparison of MPBind and the other two state-of-the-art protein–protein binding site prediction methods on two test datasets. (A) ROC curves of MPBind and PeSTo on *Test1_data* dataset. (B) Precision–recall curves of MPBind and PeSTo on *Test1_data* dataset. (C) ROC curves of MPBind, PeSTo, and ScanNet on *Test2_data* dataset. (D) Precision–recall (PR) curves of MPBind, PeSTo, and ScanNet on *Test2_data* dataset. The AUC scores under ROC and PR curves of the methods are listed in the legend of each subfigure.

The receiver operating characteristic (ROC) and precision–recall (PR) curves of MPBind and PeSTo on *Test1_data* are present in Fig. 2A and B. MPBind achieves AUROC and AUPRC scores of 0.83 and 0.54, respectively, substantially higher than 0.76 and 0.38 of PeSTo. The results in terms of Acc are provided in Table 2, available as supplementary data at *Bioinformatics* online, which also shows MPBind outperforms PeSTo. Because MPBind and PeSTo were trained, validated, and tested on the same data, the results demonstrate that MPBind is more accurate and robust than PeSTo.

We then evaluated MPBind, PeSTo, and ScanNet (Tubiana *et al.* 2022) on the *Test2_data* dataset whose proteins have less than 30% sequence identity with the proteins in their training data. ScanNet is a task-specific geometric deep learning method for predicting protein–protein binding sites, including antibody–antigen binding sites.

Among the 1452 protein chains in *Test2_data*, 1208 are involved in protein–protein interactions. Therefore, we compared the task-specific model, ScanNet, and two general (non-task-specific) models, MPBind and PeSTo, for protein binding site prediction on these 1208 chains. The results for the two critical metrics, AUROC and AUPRC, are present in Fig. 2C and D. Compared to the non-task-specific model PeSTo, MPBind achieves AUROC and AUPRC scores of 0.84 and 0.62, respectively, substantially outperforming PeSTo with scores of 0.78 and 0.48. Even when compared to the task-specific model ScanNet dedicated to protein–protein binding site prediction, MPBind still achieves better results, surpassing ScanNet by 0.01 and 0.03 in terms of AUROC and AUPRC, respectively. This improvement is largely due to MPBind’s use of much richer node features—including atom-specific characteristics, geometric descriptors, and evolutionary information derived from two PLMs (ProstT5 and ProtTrans)—which outperform the traditional MSA-based evolutionary information and the node features used in ScanNet. Furthermore, MPBind employs a more advanced EGNN architecture, further enhancing its predictive performance. The results in terms of Accuracy are provided in Table 3, available as supplementary data at *Bioinformatics* online, where MPBind achieves the highest Accuracy. Overall, these results demonstrate that MPBind achieves the state-of-the-art performance in protein–protein binding site prediction.

### 3.2 Robust generalization for DNA/RNA-, ion-, ligand-, and lipid-binding site prediction

**Figure 3.**
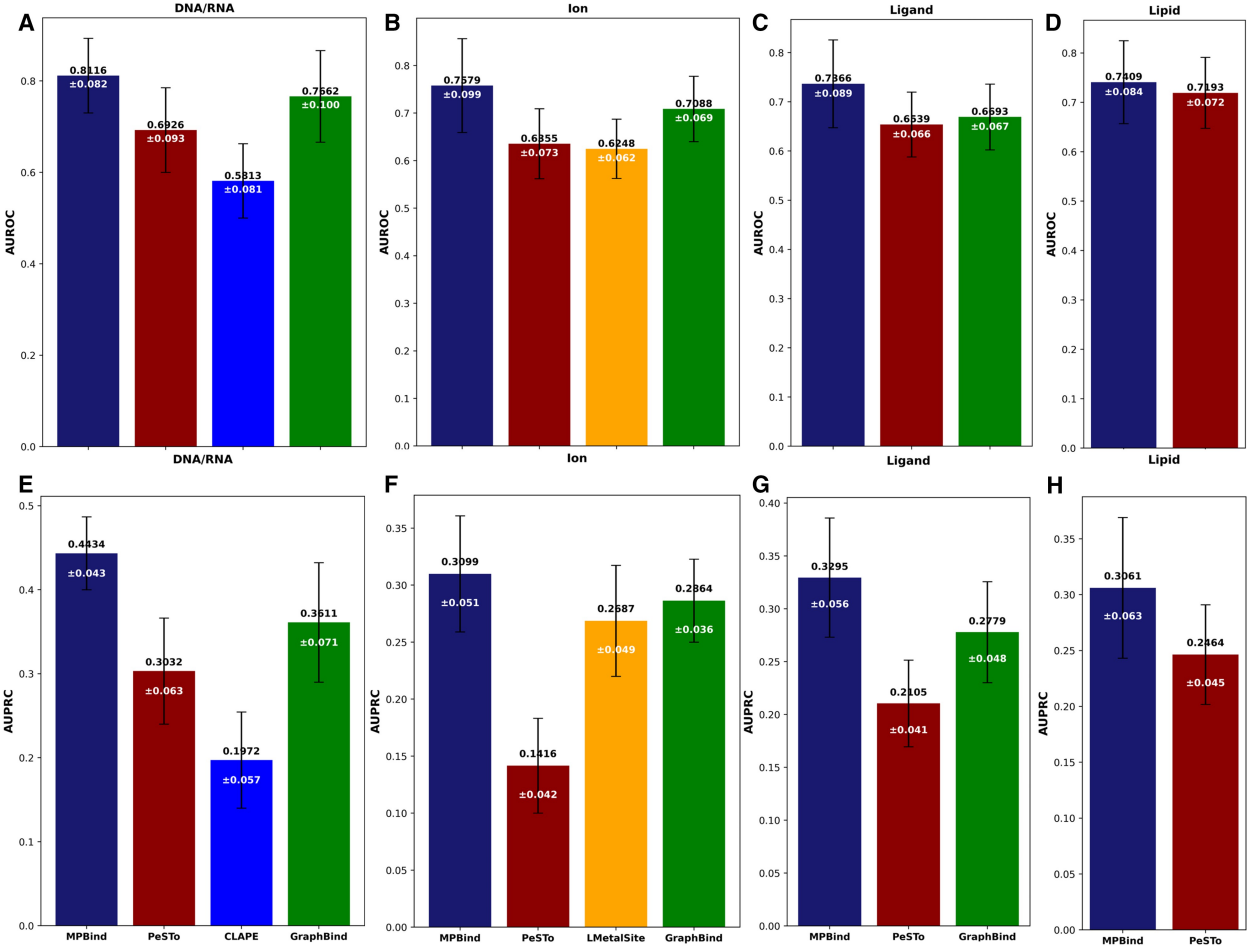
Performance comparison of MPBind with the other state-of-the-art protein–DNA/RNA-, ion-, ligand-, and lipid-binding site prediction methods on ***Test2_data***. Panels (A–D) present AUROC scores (black numbers) with corresponding standard deviations (white numbers), while panels (E and F) display AUPRC scores (black numbers) with corresponding standard deviations (white numbers) for DNA/RNA-, ion-, ligand-, and lipid-binding sites, respectively.

To evaluate MPBind’s performance in predicting the binding sites for nucleotides (DNAs or RNAs), ions, ligands, and lipids, we compared it with the same non-task-specific method, PeSTo (Krapp *et al.* 2023), along with the three top task-specific prediction methods—CLAPE (Liu and Tian 2023), LMetalSite (Yuan *et al.* 2022), and GraphBind (Xia *et al.* 2021)—on the *Test2_data* dataset. The dataset includes 75 DNA-binding chains, 48 RNA-binding chains, 406 ion-binding chains, 448 ligand-binding chains, and 39 lipid-binding chains. Notably, some chains may contain multiple binding interfaces. Following the convention in the field (Krapp *et al.* 2023), the DNA- and RNA-binding sites are treated as the same type of binding sites (nucleotide binding sites). The results in terms of the two primary metrics, AUROC and AUPRC, across four different types of binding site predictions are present in Fig. 3.

MPBind achieves AUROC scores of 0.8116, 0.7579, 0.7366, and 0.7409 for DNA/RNA-, ion-, ligand-, and lipid-binding site prediction, respectively, as shown in Fig. 3A–D. These scores are higher than those of the other non-task-specific methods [i.e. PeSTo (Krapp *et al.* 2023) for predicting binding site of any type] and task-specific methods [CLAPE (Liu and Tian 2023) for DNA/RNA-binding site prediction, LMetalSite (Yuan *et al.* 2022) for ion-binding site prediction, GraphBind (Xia *et al.* 2021) for DNA/RNA-, ion-, and ligand-binding site prediction] for all four types of binding site prediction. Compared to PeSTo, the AUROC scores of MPBind are substantially higher for DNA/RNA-, ligand-, and ion-binding site prediction and also higher for lipid-binding site prediction.

When considering the AUPRC metric, MPBind achieves scores of 0.4434, 0.3099, 0.3295, and 0.3061 for DNA-, RNA-, ion-, and lipid-binding site prediction, respectively, as shown in Fig. 3E–H. These AUPRC scores are significantly higher than those of PeSTo across all binding site types, demonstrating its superior performance. Furthermore, when compared to the leading task-specific methods—CLAPE, LMetalSite, and GraphBind—MPBind also consistently outperforms them across all types of binding site predictions.

The results shows that MPBind outperforms both the general and specialized task-specific methods for binding site prediction in terms of the two critical metrics—AUROC and AUPRC. The results in terms of Acc are detailed in Table 4, available as supplementary data at *Bioinformatics* online, also showing MPBind performs best.

Overall, the results above demonstrate that MPBind not only achieves superior results in specific types of prediction but also exhibits a remarkable level of generalization across diverse binding site types, indicating its robustness and effectiveness in accurately predicting a wide range of binding sites.

### 3.3 Five cases demonstrating MPBind’s ability to distinguish different binding interfaces

**Figure 4.**
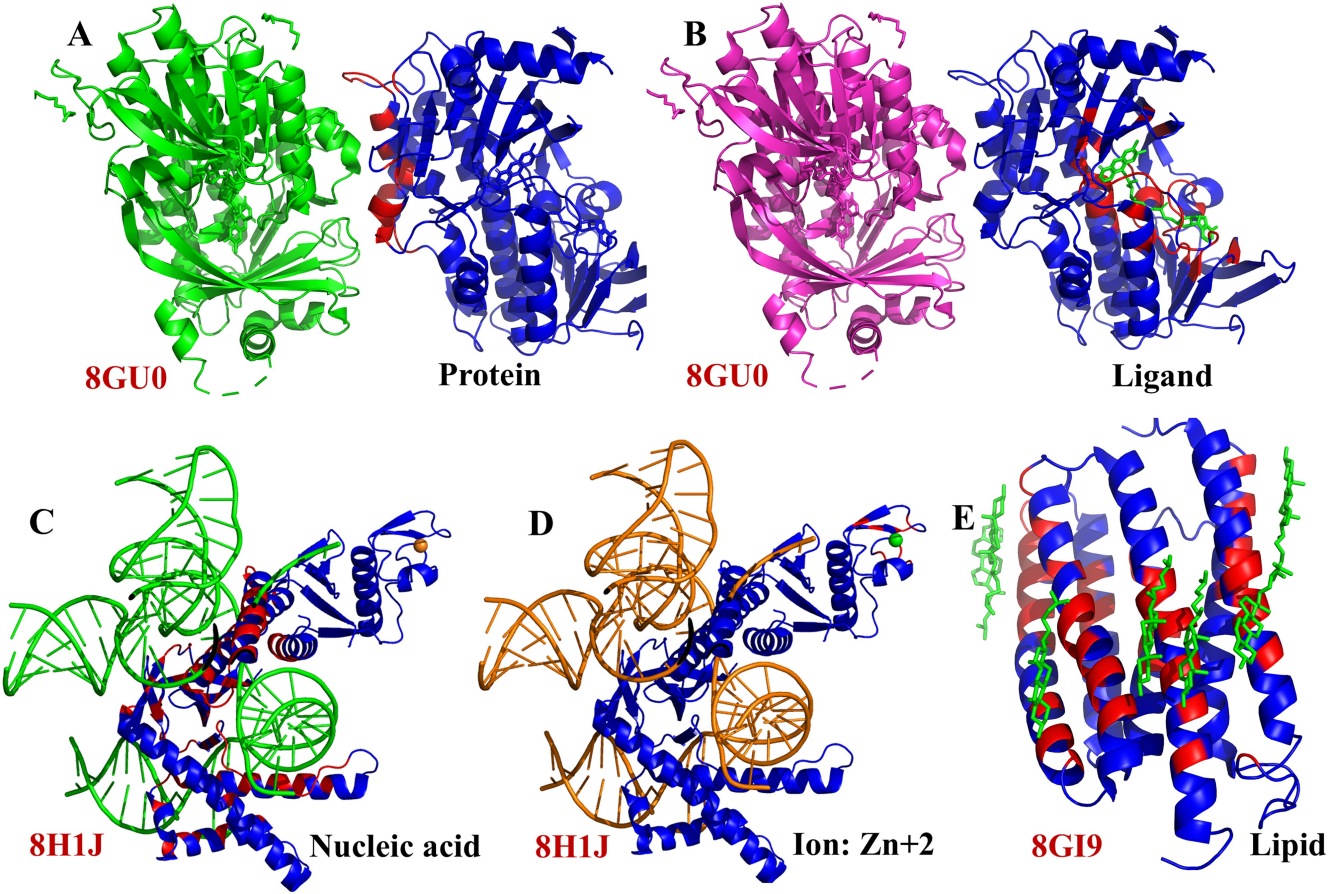
Examples of five types of binding site predictions by MPBind. The fungal halogenase RadH domain (PDB code: 8GU0) in complex with a protein (A) and a ligand, Flavin-Adenine Dinucleotide (FAD) (B). The transposon-associated TnpB enzyme (PDB code: 8H1J) in complex with nucleic acids (C) and a Zn + 2 ion (D). Cation Channelrhodopsin (PDB code: 8GI9) in complex with multiple lipids, ChoLesteRol (CLR) (E). In each subfigure, the PDB code is displayed in the bottom-left corner, the binding type is indicated in the bottom-right corner, the green region represents the partner interacting with the protein chain under consideration (for which MPBind made binding site predictions), the red region highlights the predicted binding sites at the decision threshold of 0.5, and the blue region denotes the predicted non-binding sites. In each case, some key residues binding with another molecule are correctly predicted.

To demonstrate the prediction capability of MPBind, we present five examples from the ***Test2_data*** dataset, each representing one of the five binding site prediction types, as shown in Fig. 4. The first example is the protein–protein interaction interface of the fungal halogenase RadH domain (Peh *et al.* 2023) that enables regioselective halogenation of various bioactive aromatic scaffolds. Figure 4A depicts the protein–protein interaction within the RadH domain. When this structure is processed by MPBind, it assigns a binding site probability score between [0, 1] to each residue in the right-hand chain, with the left-hand (green) chain serving as the binding partner. Twenty-three residues of the right-hand chain (448 residues in total) form a relatively large area involved in binding, which serve as true labels. MPBind assigns a score greater than 0.5–15 of these residues, identifying them as the predicted binding sites (red), while the remaining eight residues with scores smaller than 0.5 are false negatives (true binding residues missed by MPBind).

The second example is the same protein domain as in the first example, where it interacts with a ligand—flavin-adenine dinucleotide (FAD), colored in green, as shown in Fig. 4B. When the domain is analyzed by MPBind, it detects the ligand-binding sites rather accurately. Out of the 47 residues forming a large binding pocket for FAD, MPBind assigns a prediction score above 0.5–31 of these residues, predicting them as the binding sites, while the remaining 16 residues with scores under 0.5 are false negatives.

The third example is the *Deinococcus radiodurans* ISDra2 TnpB in complex with its cognate ωRNA and target DNA (Nakagawa *et al.* 2023), shown in Fig. 4C, which involves the nucleic acid binding sites. It has 362 residues in total. MPBind assigns scores above 0.5–49 out of the 97 true binding residues forming a large nucleotide binding area, while the remaining 48 residues with scores under 0.5 are false negatives.

The fourth example involves the same protein as in the third example, as shown in Fig. 4D, which also interacts with a Zn + 2 ion, represented in green. Eight residues form a binding pocket for the ion, identified as labels. MPBind predicts seven of these eight true binding residues as ion-binding sites with a score above 0.5, while the remaining one residue is missed.

The last example is channelrhodopsin 1 from *Hyphochytrium catenoides*, which is a light-activated channel employed for optogenetic silencing of mammalian neurons, as shown in Fig. 4E. It consists of 240 residues and binds with multiple lipids. MPBind predicts 12 out of the 40 true lipid-binding residues as binding sites with scores above 0.5 but misses the remaining 28 residues.

The examples above show that MPBind can accurately identify some key binding residues and the location of interaction interfaces for five types of binding partners, even though it cannot predict all the binding residues at a decision threshold of 0.5. Lowering the threshold can predict more binding residues at the expense of a higher false positive rate.

### 3.4 Comparing binding site prediction of using predicted and true protein structures

**Figure 5.**
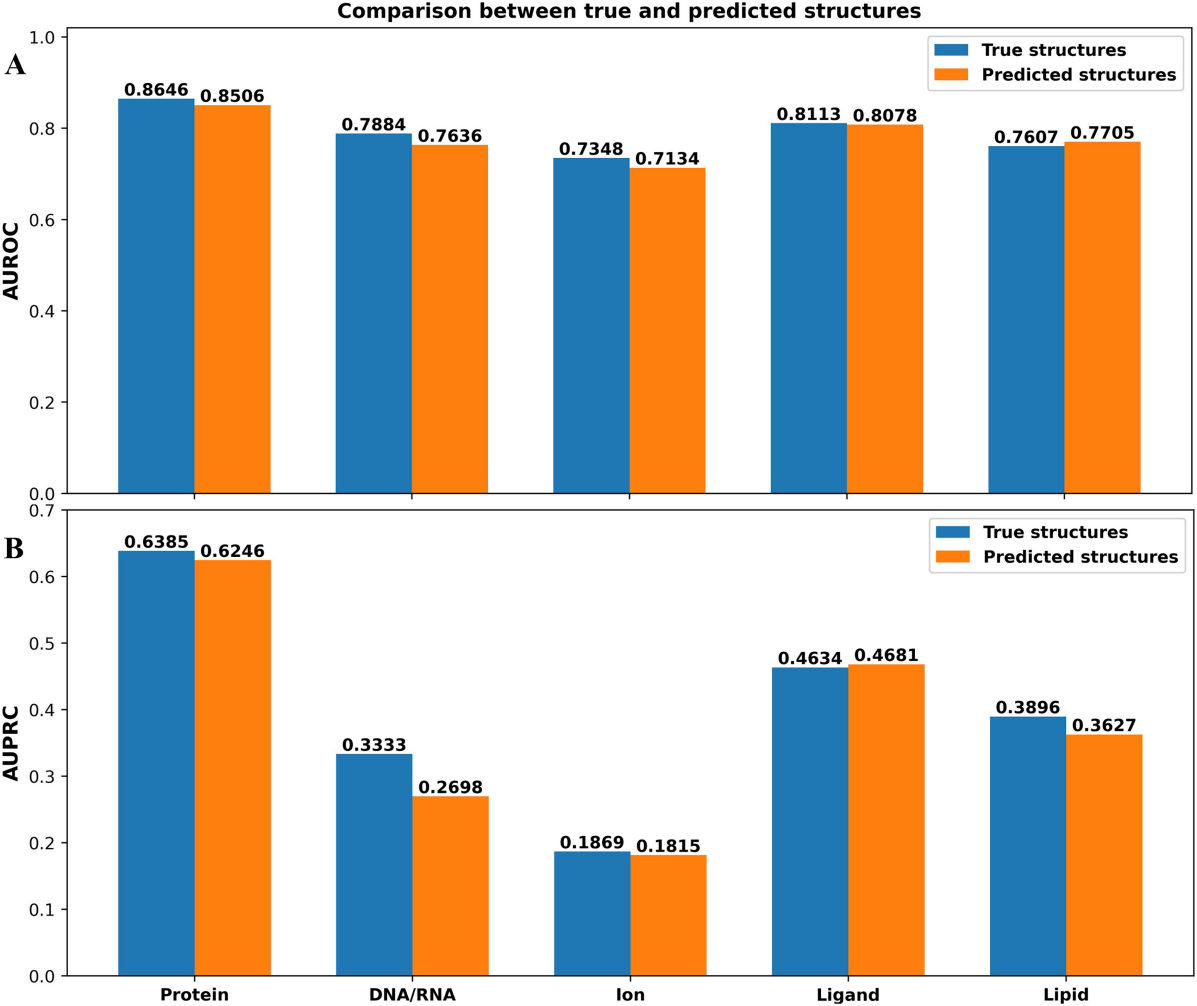
Performance comparison of using true and AlphaFold 3-predicted structures with MPBind for predicting protein–protein-, DNA/RNA-, ion-, ligand-, and lipid-binding sites on 100 randomly selected protein chains. (A) AUROC scores. (B) AUPRC scores.

MPBind was trained with true protein structures as input. To investigate how well it can predict binding sites from predicted protein structures, we randomly selected 20 protein chains for each of the five kinds of binding sites from ***Test2_data*** dataset (i.e. 100 protein chains in total) and applied AlphaFold 3 (Abramson *et al.* 2024) to predict protein structures for them. The predicted protein structures were used for MPBind to predict binding sites. The results of using predicted protein structures are compared with those of using true structures in Fig. 5 and Fig. 1, available as supplementary data at *Bioinformatics* online. In terms of AUROC (Fig. 5), MPBind performs slightly better on true structures for protein–protein-, DNA/RNA-, ion-, and ligand-binding site predictions but slightly worse for protein–lipid-binding site prediction than on the corresponding AlphaFold 3-predicted structures. One possible reason for this is that the quality of AlphaFold-predicted structures is very close to that of the true experimental structures so that they lead to similar prediction performance. And MPBind may not be very sensitive to the minor change in the accuracy of input structures. Moreover, even though the experimental structures are slightly more accurate, they sometime are incomplete and miss the coordinates for some residues due to experimental constraints, while the AlphaFold-predicted structures are always complete and contain structural information for all the residues. If the advantage caused by the completeness of input structures outweighs the disadvantage associated with their slightly lower accuracy in some case, using AlphaFold-predicted structures may lead to slightly better prediction accuracy. This phenomenon is consistent with the surprising finding in the recent community-wide Critical Assessment of Protein Structure Prediction (CASP16) (Gilson *et al.* 2025) that using experimental protein structures as input did not improve protein–ligand-binding affinity prediction over using AlphaFold-predicted structures.

Similarly, in terms of AUPRC (Fig. 5), MPBind shows better performance on true structures than on the corresponding AlphaFold 3-predicted structures for four binding site types including protein–protein-, DNA/RNA-, ion-, and lipid-binding sites, but the difference is marginal except for DNA/RNA-binding sites, indicating the DNA/RNA-binding prediction is more sensitive to the quality of input structures. For protein–ligand-binding site prediction, MPBind performs slightly better on AlphaFold 3-predicted structures than on true structures. In terms of Accuracy (Fig. 1, available as supplementary data at *Bioinformatics* online), the performance of using true and predicted protein structures as input for MPBind is almost the same. Taken together, the performance of using MPBind with predicted and true protein structures is very similar, indicating that MPBind can be applied to most proteins that have no true (experimental) structures. It also demonstrates that the AlphaFold3-predicted structures are accurate enough for binding site prediction.

### 3.5 Analysis of predicted binding sites in the human proteome

The human proteome is of great interest to researchers. Here, we analyze the binding sites predicted by MPBind for the proteins in the human proteome with functional annotations in UniProt (UniProt Consortium 2019). We retrieved all predicted human protein structures, totaling 23 391 structures, from the AlphaFold-European Bioinformatics Institute database (AlphaFoldDB) (Varadi *et al.* 2024). We selected 7435 high-quality structures with a predicted local distance difference test (pLDDT) score of >70.0 and a predicted alignment error (PAE) of <10.0 for further analysis. The residue-annotated features for each selected structure were downloaded from the UniProt database (UniProt Consortium 2019).

**Figure 6.**
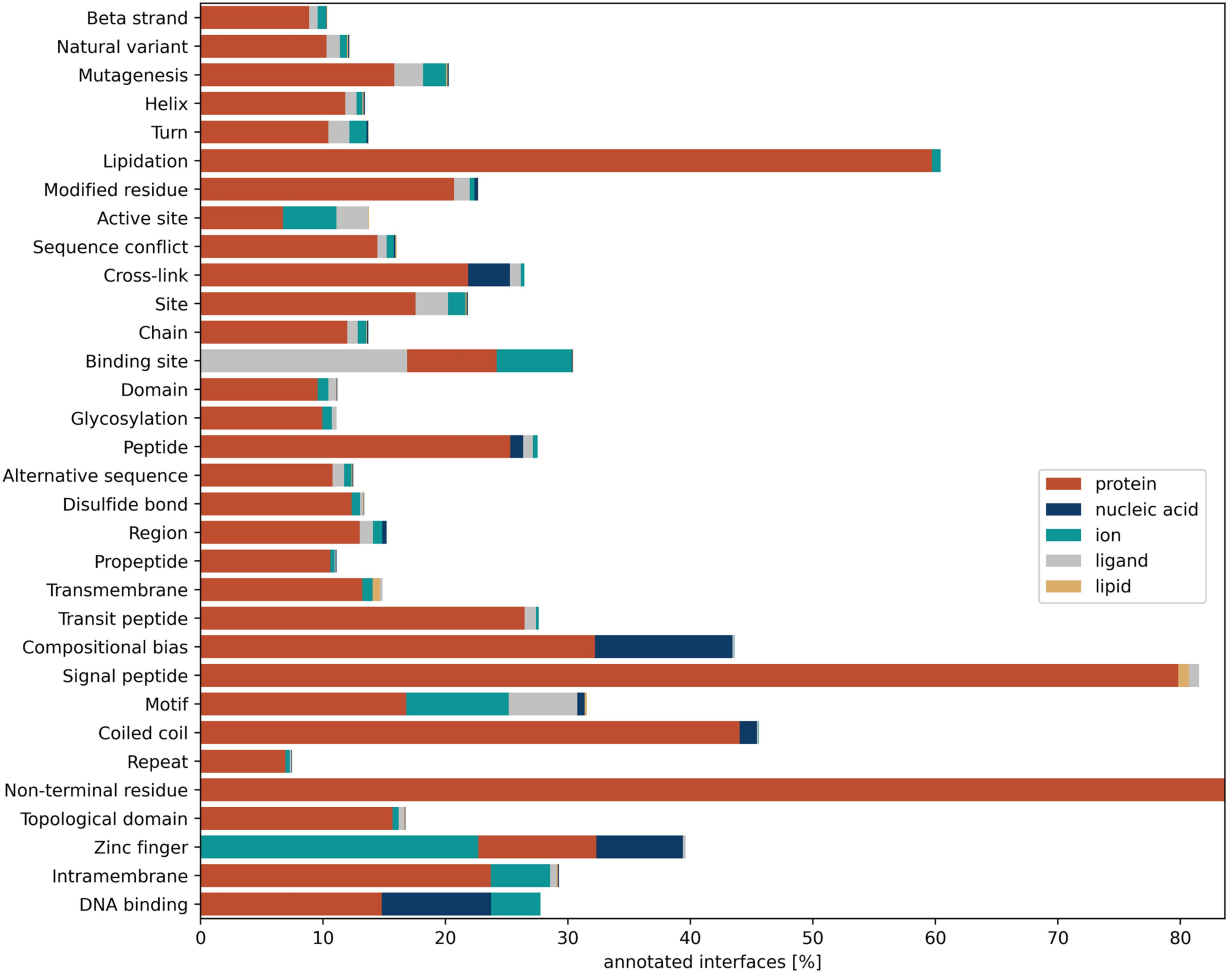
Proportion of proteins with specific annotated features in UniProt that are predicted by MPBind to have each type of binding site within the designated sequence region of the features.

After processing all the selected structures with MPBind, we mapped the predicted binding sites to the corresponding UniProt-annotated residue-level features. Among all the annotated residue-level features, for proteins with each specific residue-level feature, we calculated the proportion of the proteins whose feature-containing sequence regions overlapped with the binding sites predicted by MPBind for each binding type. The overlap was defined at the per-residue level. For example, if a UniProt-annotated feature spanned residues 1–39 and MPBind predicted binding sites at residues 21–45, the overlap was residues 21–39. Even a single overlapping residue qualified the protein as containing both the annotated feature and the predicted binding site type. For instance, as shown in Fig. 6, MPBind predicts that over 80% of proteins with annotated non-terminal residues have protein–protein interaction interfaces (binding sites) within those regions. This indicates a strong prevalence of non-terminal residues in protein–protein interactions, consistent with previous findings (Martin *et al.* 2016). In another example, MPBind predicts more than 20% of proteins with the annotated zinc finger feature have predicted protein–ion-binding sites in those regions, which is consistent with the function of zinc fingers. Zinc fingers are well-known structural motifs that coordinate zinc ions to stabilize the fold of proteins, enabling them to interact with DNA, RNA, or other proteins (Pavletich and Pabo 1991, Laity *et al.* 2001). This ion coordination is crucial for the proper functioning of zinc finger proteins in gene regulation, DNA repair, and transcriptional control. Therefore, MPBind’s ability to identify ion-binding sites in these regions reflects its biological relevance and accuracy in capturing functionally important interactions. Moreover, a large proportion of proteins with the binding site feature are predicted to interact with ligands. The results demonstrate that MPBind binding site predictions can align well with the expected functional roles of the residues in the UniProt. Therefore, one of them can be used to infer another.

**Figure 7.**
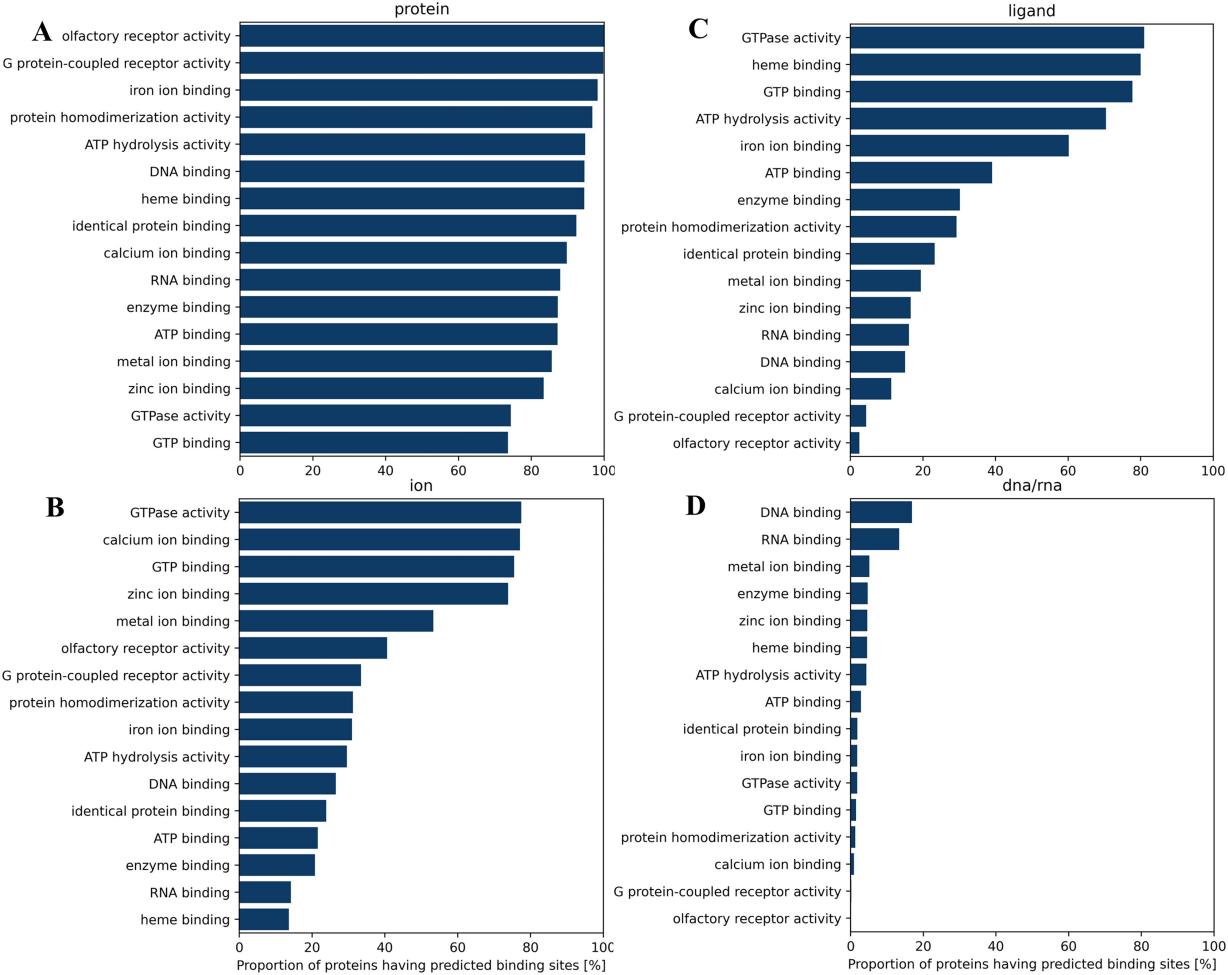
The proportion of proteins annotated with some gene ontology (GO) molecular function (MF) terms in UniProt that are associated with each of four types of binding sites predicted by MPBind. The GO terms are ordered according to the percentage. (A) Protein-binding sites, (B) ion-binding sites, (C) ligand-binding sites, and (D) DNA/RNA-binding sites. For instance, over 90% of entries annotated with olfactory receptor activity are associated with the predicted protein–protein binding sites in (A), whereas almost none are linked to the predicted DNA/RNA-binding sites in (D).

We further analyze how each type of predicted binding site (protein, ion, ligand, and DNA/RNA) is related to the overall function of proteins in terms of gene ontology (GO) terms, including molecular function (MF), biological process (BP), and cellular component (CC). GO terms were selected based on the total number of occurrences of each specific term across all types of binding sites, using a threshold of more than 200 occurrences. We calculated the proportion of proteins with a specific GO term that have binding sites predicted by MPBind for each type of binding (Fig. 7 for protein-, DNA/RNA-, ion-, and ligand-binding sites and Fig. 2, available as supplementary data at *Bioinformatics* online for lipid-binding sites). An interesting phenomenon, as shown in Fig. 7 and Fig. 2A, available as supplementary data at *Bioinformatics* online, is that generally a higher proportion of proteins annotated with the MF GO terms are associated with protein-, ligand-, and ion-binding sites predicted by MPBind than with predicted DNA/RNA-binding sites. A similar trend is observed for BP and CC GO terms, as shown in Figs 2–4, available as supplementary data at *Bioinformatics* online. One possible reason is that DNA/RNA-binding site-containing protein chains are generally less frequent than the other types of binding sites.

Additionally, some proteins annotated with MF GO terms (e.g. olfactory receptor activity) have multiple types of binding sites simultaneously (e.g. both protein- and ion-binding sites for olfactory receptor activity), as shown in Fig. 7A and B. This is biologically plausible because olfactory receptors are G protein-coupled receptors that detect odorant molecules via extracellular ligand binding while also relying on ion interactions to initiate and amplify neuronal signaling (Buck and Axel 1991, Firestein 2001). In addition, the presence of protein–protein interaction sites may reflect the need for receptor dimerization or association with intracellular signaling partners, processes that are essential for proper olfactory function (Park *et al.* 2008). Furthermore, as expected, the GO term with the highest proportion of proteins having predicted DNA-binding sites is the DNA-binding GO term (Fig. 7D). Interestingly, a significant portion of the proteins with the DNA-binding GO term also have the predicted ion-binding sites (Fig. 7B), indicating that ion binding may be important for DNA binding. The results show that predicted binding sites can be used to infer protein function and *vice versa*.

### 3.6 An ablation study of different features in MPBind

We investigated the contribution of each type of node feature to the performance of MPBind on the *Test2_data* dataset. Starting from the four types of features (ProtTrans sequence embedding, DSSP features, Atomic Residue features, and Geometric Residue features) with AUROC = 0.77 and AUPRC = 0.54 (the first row in Table 5, available as supplementary data at *Bioinformatics* online), we added one of the two kinds of embeddings generated by a PLM—ProstT5 (i.e. ProstT5(AA): amino acid sequence embedding and ProstT5(3Di): ProstT5 structural alphabet embedding), respectively. Adding ProstT5(AA) substantially increased the AUROC and AUPRC to 0.84 and 0.62 (the second row in Table 5, available as supplementary data at *Bioinformatics* online), while adding ProstT(3Di) did not affect AUROC and only slightly increased AUPRC to 0.55 (the third row in Table 4, available as supplementary data at *Bioinformatics* online). This is likely because the coarse-grained residue–residue geometry representation of 3Di lacks fine-grained atomic details often required for more accurate binding site recognition and/or because MPBind already incorporates rich atomic, geometric, and sequence-derived features, making the additional 3Di signal potentially some redundant (Fout *et al*. 2017, Gainza, *et al.* 2020). Therefore, we chose to only use ProstT5(AA) features with the other four types of features as the start point in the following three feature deletion experiments. We tried to delete one of the three types (DSSP features, Atomic Residue features, and Geometric Residue features) in each of the three separate experiments (the fourth, fifth, and sixth rows in Table 5, available as supplementary data at *Bioinformatics* online). Deleting any one of them decreased the performance, indicating that each of them is useful, while the impact of the DSSP features is higher than the Atomic Residue features, and the Geometric Residue features are least important. The ablation study demonstrates that using ProtTrans, ProstT5 (AA), DSSP, Atomic Residue, and Geometric Residue features together yields the best results. The combination of the five types of features is used in the final version of MPBind.

## 4 Conclusion

In this study, we developed a new multitasking method (MPBind) of using PLMs and an EGNN to integrate both protein sequence and structure data to predict five types of protein binding sites. A graph with some new node and edge features are designed to capture the sequence, structural, and geometrical features of input proteins to be processed by MPBind. MPBind can not only predict a broad spectrum of protein binding sites interacting with proteins, DNA, RNA, ligands, ions, and lipids, but also outperforms other general or task-specific binding site prediction methods. Additional analysis of MPBind predicted binding sites for the human proteins show that they are consistent with the annotated functional features of the proteins in the UniProt. The results demonstrate that MPBind can be used to predict the binding sites of unannotated proteins and facilitate their functional and structural study.

## Supplementary Material

btaf589_Supplementary_Data

## Data Availability

The source code of MPBind is available at the GitHub repository: https://github.com/jianlin-cheng/MPBind. The original protein structures used to create the datasets can be downloaded from the PDB by a script provided at the GitHub repository: https://github.com/jianlin-cheng/MPBind. The scripts for generating training and test datasets from the structures are provided at the GitHub repository. The predicted Human Proteome interfaces can be downloaded from https://zenodo.org/records/17400473.
