# Supplementary material for: MPBind: a multitask protein binding site predictor using protein language models and equivariant GNNs
Source: Bioinformatics. 2025 Oct 24;41(11):btaf589. doi: 10.1093/bioinformatics/btaf589 (PMC12631785; doi:10.1093/bioinformatics/btaf589)
Supplement: btaf589_Supplementary_Data [file btaf589_supplementary_data.docx]

**Supplementary Materials for**

**MPBind: A Multitask Protein Binding Site Predictor Using Protein Language Models and Equivariant GNNs**

**S1. Supplementary Methods**

**S.1.1 Feature Extraction**

Both the first and second types of node features are based on the embedding information extracted from protein sequences by Protein Language Models (PLMs) (Elnaggar, et al., 2021; Heinzinger, et al., 2024; Yuan, et al., 2022), as they have shown better performance in various protein property prediction problems than traditional approaches of using multiple sequence alignment (MSA) as input. Two pre-trained PLMs: ProtTrans (Elnaggar, et al., 2021) and ProstT5 (Heinzinger, et al., 2024), which were trained on different datasets and offer complementary insights, are incorporated into our model to extract residue information. ProtTrans is based on a single modality – protein sequence, while ProstT5 is a multimodal PLM considering both sequence and structure. Each PLM provides residue features of size $L\times1024$ for a protein of length *L*, where 1024 denotes the dimension of the features per residue.

The third type of node feature is secondary structures and solvent accessibility, extracted using DSSP (Kabsch and Sander, 1983; Touw, et al., 2015) from the 3D structures. This approach generates residue-level node features of size $L\times9$ for a protein of length *L*, where the 9 dimensions of the features per residue include the relative solvent accessibility (the solvent accessible surface of a residue normalized by the maximum solvent accessible surface area of its corresponding amino acid type) and the one-hot encoding of the eight possible secondary structure types.

The fourth type of node feature is the atomic features for each residue. Each residue contains various atoms, and each atom within a specific residue has several properties, such as atomic mass, whether it is a side-chain atom, whether it is part of a ring, the amount of electronic charge, the number of hydrogen atoms bonded to it, and the length of the van der Waals radius. We select these six properties of each atom in a residue as the atomic features. However, since the number of atoms varies across different types of residues, we average the atomic properties within each residue, resulting in atomic node features of size $L\times6$ for an input structure with *L* residues.

The final type of node feature is geometric node features, which are the same as the ones used in GPSite. Each atom in a residue has a unique position in a protein structure, and the side-chain atoms of a residue can be centralized by averaging their positions, denoted as R. Therefore, the position of each residue can be represented by the x, y, and z coordinates of four main backbone atoms ($N$, $C$, $C_{a}$, $O$) and *R*. Using this position information, we can calculate the bond and torsion angles, intra-residue distances, and relative directions of other inner atoms (i.e., $N$, $C$, $O$, and R) to $C_{a}$ atom respectively, which form the geometric information for each residue (i.e., geometric residue). These resulting geometric node features have a size of $L\times184$ for a protein structure with *L* residues.

**S2. Supplementary Tables and Figures**

| **Table S1.** Statistics of five binding site types in the two test datasets. | | | | | | |
| --- | --- | --- | --- | --- | --- | --- |
| **Binding site type** | | **Protein** | **DNA/RNA** | **Ion** | **Ligand** | **Lipid** |
| ***Test1_data*** | **#** of Chains | 32940 | 393 | 11905 | 16278 | 161 |
|  | Ratio of Binding Residues | 0.196 | 0.087 | 0.045 | 0.073 | 0.058 |
|  | **#** of Binding Residues | 1380990 | 7713 | 129305 | 287101 | 3627 |
|  | **#** of Total Residues | 7036334 | 88336 | 2852542 | 3949163 | 62652 |
| ***Test2_data*** | **#** of Chains | 1208 | 97 | 406 | 448 | 39 |
|  | Ratio of Binding Residues | 0.214 | 0.134 | 0.036 | 0.078 | 0.110 |
|  | **#** of Binding Residues | 52888 | 3581 | 3814 | 9086 | 956 |
|  | **#** of Total Residues | 247255 | 26737 | 106496 | 116457 | 8661 |

| **Table S2**. Five types of node features with corresponding feature dimensions for a given protein of length *L.* | | | | | | |
| --- | --- | --- | --- | --- | --- | --- |
| Node feature type | ProtTrans | ProstT5 | DSSP | Atomic | Geometric | Total |
| Feature dimension | $L\times1024$ | $L\times1024$ | $L\times9$ | $L\times6$ | $L\times184$ | $L\times2247$ |

| **Table S3:** Performance comparison of MPBind and two protein-protein binding site prediction methods across two test datasets. Bold fond denotes the best result. | | |
| --- | --- | --- |
| **Method** | ***Test1_data*** | ***Test2_data*** |
|  | **Accuracy** | **Accuracy** |
| MPBind | **0.8468** | **0.8271** |
| PeSTo | 0.7905 | 0.7559 |
| ScanNet | --- | 0.7902 |

| **Table S4:** Performance comparison of MPBind and state-of-the-art protein-DNA/RNA, ion, ligand, and lipid binding site prediction methods on ***Test2_data*** dataset. | | |
| --- | --- | --- |
| **Binding site type** | **Method** | ***Test2_data*** |
|  |  | **Accuracy** |
| **DNA/RNA** | MPBind | **0.9902** |
|  | PeSTo | 0.9372 |
|  | CLAPE | 0.8825 |
|  | GraphBind | 0.8773 |
| **Ion** | MPBind | **0.9875** |
|  | PeSTo | 0.9874 |
|  | LMetalSite (Ca^2+^, Mg^2+^, Mn^2+^, Zn^2+^) | 0.9673 |
|  | GraphBind (Ca^2+^, Mg^2+^, Mn^2+^) | 0.9666 |
| **Ligand** | MPBind | **0.9761** |
|  | PeSTo | 0.9744 |
|  | GraphBind (ATP, HEME) | 0.9203 |
| **Lipid** | MPBind | **0.9972** |
|  | PeSTo | 0.9505 |

| **Table S5.** Contribution of different node feature types to the performance of MPBind on the *Test2_data* dataset in terms of AUROC and AUPRC. Bold font denotes the best result. | | | | | | | |
| --- | --- | --- | --- | --- | --- | --- | --- |
| ProtTrans | ProstT5 (AA) | ProstT5 (3Di) | DSSP | Atomic  Residue | Geometric  Residue | AUROC | AUPRC |
| ✅ | ❌ | ❌ | ✅ | ✅ | ✅ | 0.77 | 0.54 |
| ✅ | ✅ | ❌ | ✅ | ✅ | ✅ | **0.84** | **0.62** |
| ✅ | ❌ | ✅ | ✅ | ✅ | ✅ | 0.77 | 0.55 |
| ✅ | ✅ | ❌ | ❌ | ✅ | ✅ | 0.78 | 0.57 |
| ✅ | ✅ | ❌ | ✅ | ❌ | ✅ | 0.80 | 0.59 |
| ✅ | ✅ | ❌ | ✅ | ✅ | ❌ | 0.81 | 0.60 |
| ✅ = feature included  ❌ = feature removed | | | | | | | |


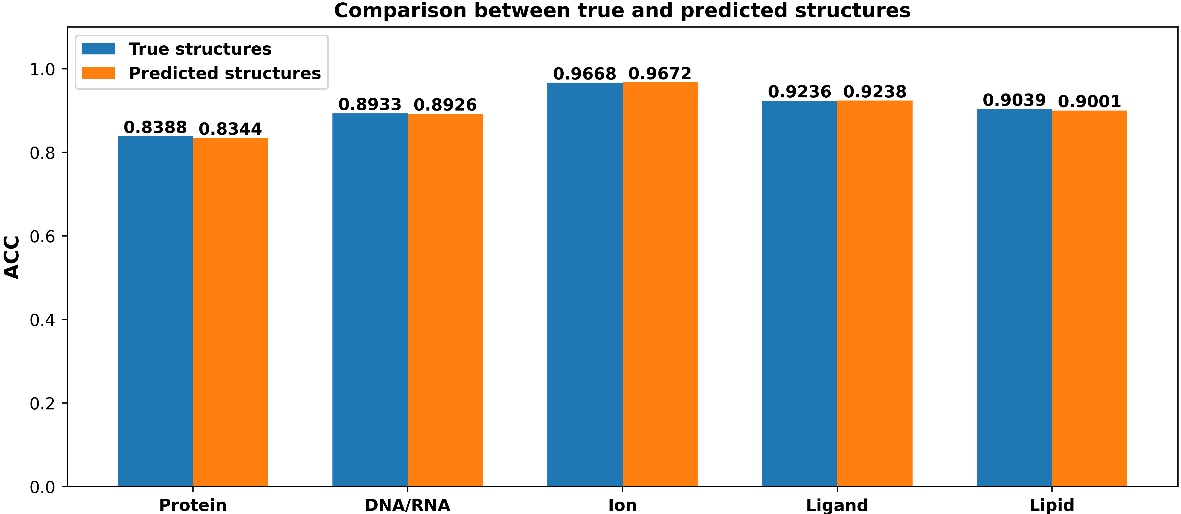


**Fig. S1:** Comparison of Accuracy (Acc) of using true and AlphaFold 3-predicted structures with MPBind for predicting protein-protein, DNA/RNA, ion, ligand, and lipid binding sites on 100 randomly selected chains from the ***Test2_data*** dataset.


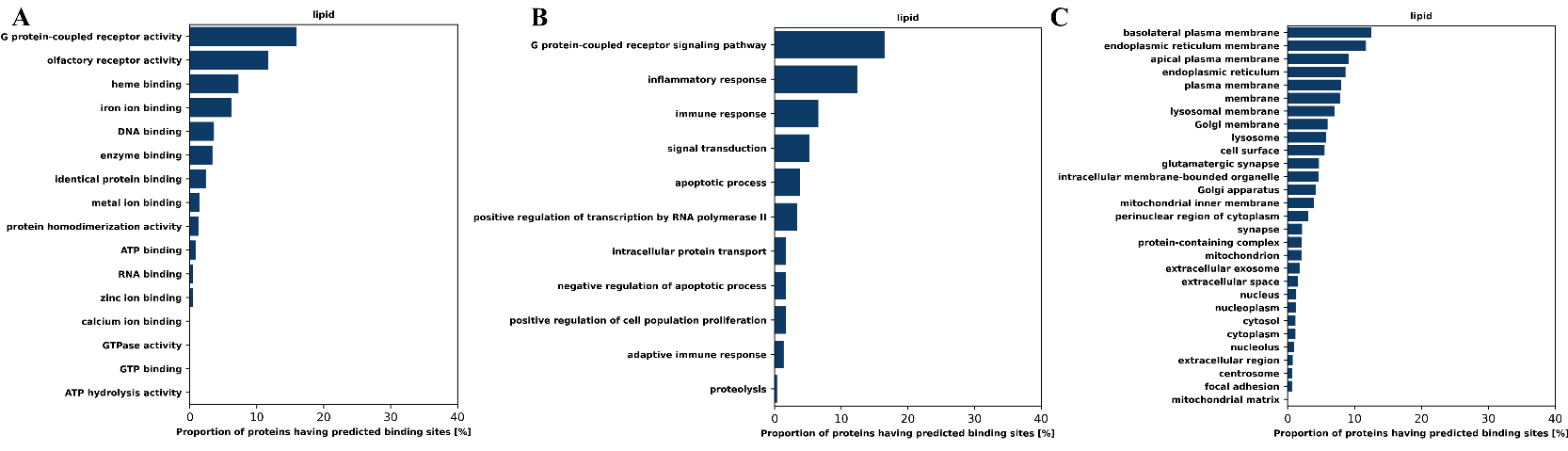


**Fig. S2:** The proportion of proteins with some Gene Ontology (GO) terms from UniProt that are associated with lipid binding sites predicted by MPBind. The GO terms are ordered according to the percentage. (**A**) molecular function (MF) terms, (**B**) biological process (BP) terms, and (**C**) cellular component (CC) terms.


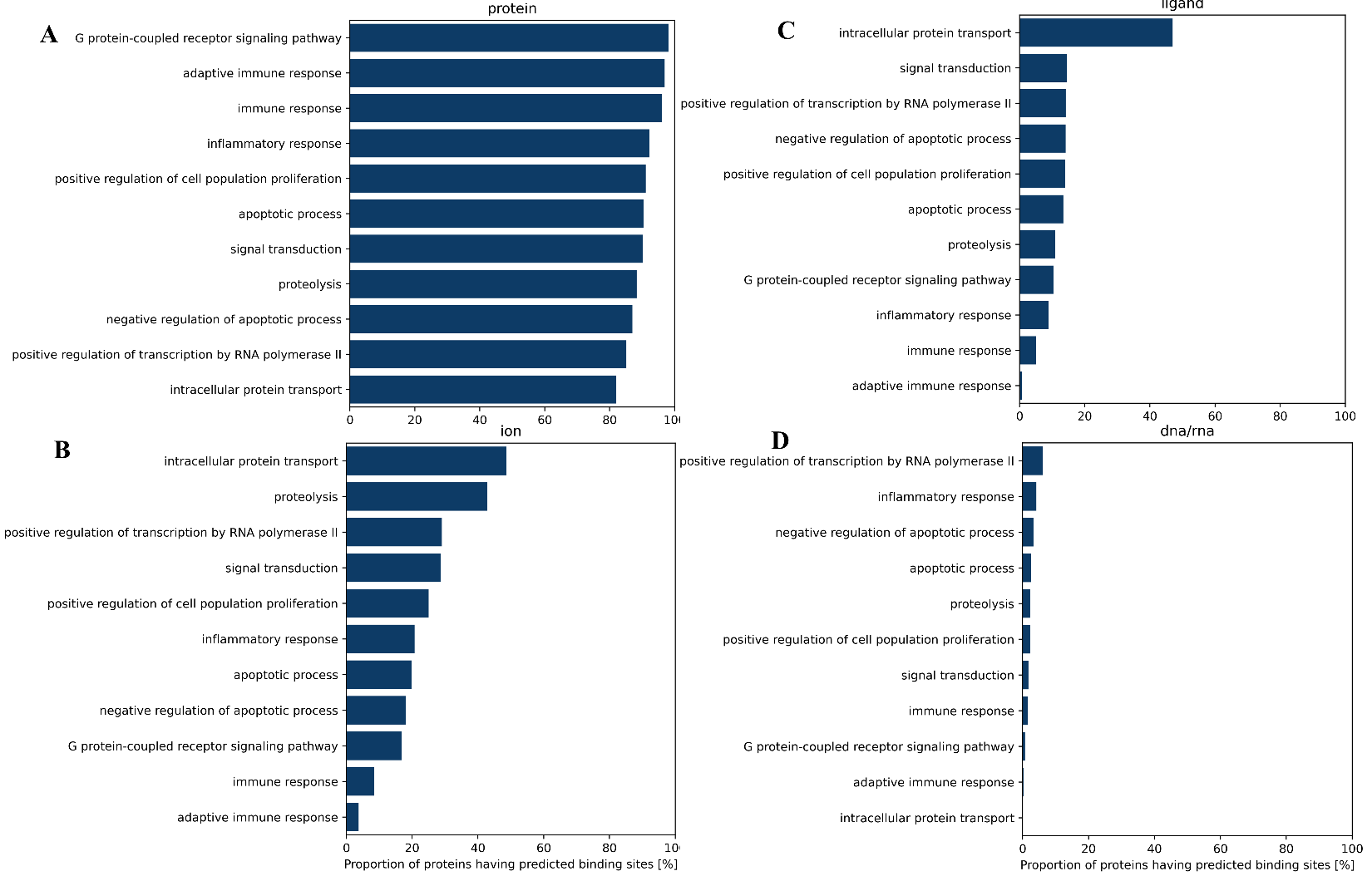


**Fig. S3:** The proportion of proteins with some biological process GO terms from UniProt that are associated with four different types of binding sites predicted by MPBind. The GO terms are ordered according to the percentage. (**A**) protein binding sites, (**B**) ion binding sites, (**C**) ligand binding sites, and (**D**) DNA/RNA binding sites.


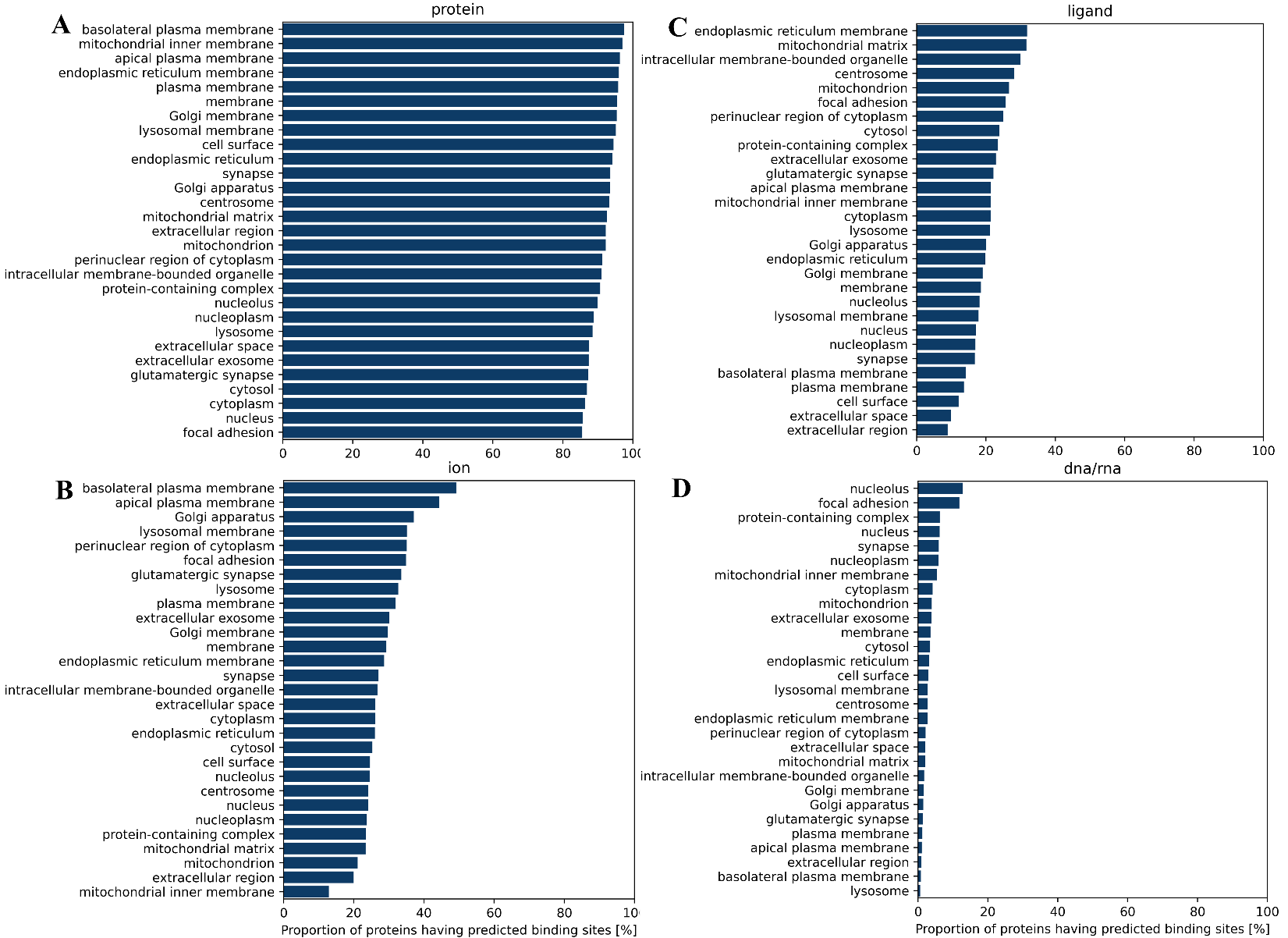


**Fig. S4:** The proportion of proteins with some cellular component GO terms from UniProt that are associated with distinct types of binding sites predicted by MPBind. The GO terms were ordered according to the percentage. (**A**) protein binding sites, (**B**) ion binding sites, (**C**) ligand binding sites, and (**D**) DNA/RNA binding sites.

**References**

Elnaggar, A.*, et al.* Prottrans: Toward understanding the language of life through self-supervised learning. *IEEE transactions on pattern analysis and machine intelligence* 2021;44(10):7112-7127.

Heinzinger, M.*, et al.* Bilingual language model for protein sequence and structure. *NAR Genomics and Bioinformatics* 2024;6(4):lqae150.

Kabsch, W. and Sander, C. Dictionary of protein secondary structure: pattern recognition of hydrogen‐bonded and geometrical features. *Biopolymers: Original Research on Biomolecules* 1983;22(12):2577-2637.

Touw, W.G.*, et al.* A series of PDB-related databanks for everyday needs. *Nucleic acids research* 2015;43(D1):D364-D368.

Yuan, Q.*, et al.* Alignment-free metal ion-binding site prediction from protein sequence through pretrained language model and multi-task learning. *Briefings in bioinformatics* 2022;23(6):bbac444.
